# Association of Dietary α-Carotene and β-Carotene Intake with Low Cognitive Performance in Older Adults: A Cross-Sectional Study from the National Health and Nutrition Examination Survey

**DOI:** 10.3390/nu15010239

**Published:** 2023-01-03

**Authors:** Qiya Zhong, Wen Sun, Yao Qin, Huadong Xu

**Affiliations:** 1 School of Public Health, Sun Yat-sen University, Guangzhou 510080, China; 2 School of Public Health, Hangzhou Medical College, Hangzhou 310013, China

**Keywords:** α-carotene, β-carotene, cognitive function, cross-sectional study

## Abstract

This study aims to examine the relationships of dietary α-carotene and β-carotene intake with cognitive function. The data were selected from the National Health and Nutrition Examination Survey (NHANES) 2011–2014. A total of 2009 participants were included in this analysis. Dietary α-carotene and β-carotene intake were averaged by two 24-h dietary recalls. The Consortium to Establish a Registry for Alzheimer’s Disease Word Learning subset (CERAD W-L), Animal Fluency Test (AFT), and Digit Symbol Substitution Test (DSST) were used to evaluate cognitive function. Logistic regression and restricted cubic spline models were applied to explore the associations of dietary α-carotene and β-carotene intake with cognitive performance. After adjusting for all confounding factors, compared with individuals in the lowest quartile of β-carotene dietary intake, those in the highest quartile had lower risks of both CERAD W-L decline [odds ratio (OR) = 0.63, 95% confidence interval (CI): 0.44–0.90] and AFT decline (OR = 0.66, 95% CI: 0.47–0.94). In addition, the third quartile of β-carotene dietary intake had a significantly decreased risk of lower DSST (OR = 0.67, 95% CI: 0.48–0.83). Compared with the lowest quartile of α-carotene intake, the OR of AFT decline in the highest intake quartile was 0.66 (95% CI: 0.46, 0.94). For males, both dietary α-carotene and β-carotene intake were associated with a decreased risk of AFT decline (OR = 0.42, 95% CI: 0.25–0.71; OR = 0.51, 95% CI: 0.30–0.85, respectively). For females, dietary α-carotene intake was associated with a decreased risk of CERAD W-L decline (OR = 0.55, 95% CI: 0.33–0.91) and dietary β-carotene intake was associated with decreased risks of both CERAD W-L and AFT decline (OR = 0.37, 95% CI: 0.21–0.64; OR = 0.58, 95% CI: 0.37–0.91, respectively). Our results suggested that higher dietary α-carotene and β-carotene intake had inverse effects on cognitive function decline among older adults.

## 1. Introduction

With the increased life expectancy around the world, the number of elderly people with cognitive decline has been escalating, causing a burden for their families and governments. The decline in cognitive function is associated with various factors [1], including normal aging processes and neurological diseases. However, without any prevention measures to delay cognitive function decline, the decline in cognitive function will gradually develop into mild cognitive impairment (MCI) and Alzheimer’s disease [2]. The process of Alzheimer’s disease is irreversible, and medical treatment for this disease is still limited. According to statistics in 2021, there were approximately 6.2 million Alzheimer’s patients in America [3], and this number was estimated to rise to 13.8 million by the mid-century. Deaths resulting from Alzheimer’s disease increased by more than 145% between 2000 and 2019, which is far more serious than death from stroke, cancer, and heart disease [3]. Thus, it is important to explore the risk factors of Alzheimer’s disease and other cognitive impairments.

Previous studies have shown that lack of physical activity [4], obesity [5], low education level [6], smoking [7], and lack of nutrition [8], such as vitamin B12 and metals [9], are risk factors associated with cognitive function decline. The underlying mechanism of vitamin B12 on cognitive function is related to the activation of methylation reactions in the brain [10]. According to previous studies, vitamin A, an antioxidant in the central nervous system [11], also participates in cognitive function decline in older people [12]. Both α-carotene and β-carotene can be transformed into retinol, which will be converted into a long-chain fatty acid ester that is the main precursor of vitamin A in the human body [13,14]. Thus, α-carotene and β-carotene may have similar effects on neurocognitive decline. Some previous studies showed that higher levels of α-carotene and β-carotene in the plasma were associated with better cognitive function [15,16]. However, the relationships of dietary α-carotene and β-carotene consumption with cognitive function have not been well explored.

Therefore, we used the National Health and Nutrition Examination Survey (NHANES) to explore the association of dietary α-carotene and β-carotene intake with cognitive function in elderly people. In addition, we also investigated the dose-response relationships of dietary α-carotene and β-carotene intake with cognitive function decline.

## 2. Materials and Methods

### 2.1. Study Population

As a cross-sectional survey, the NHANES was administered by the Centers for Disease Control and Prevention (CDC), aiming to estimate the health and nutritional status of the U.S. population [17]. The protocols in the NHANES were approved by the Review Board of the National Center for Health Statistics Ethics (NCHS). All the participants provided informed consent before the survey.

**Figure 1:**
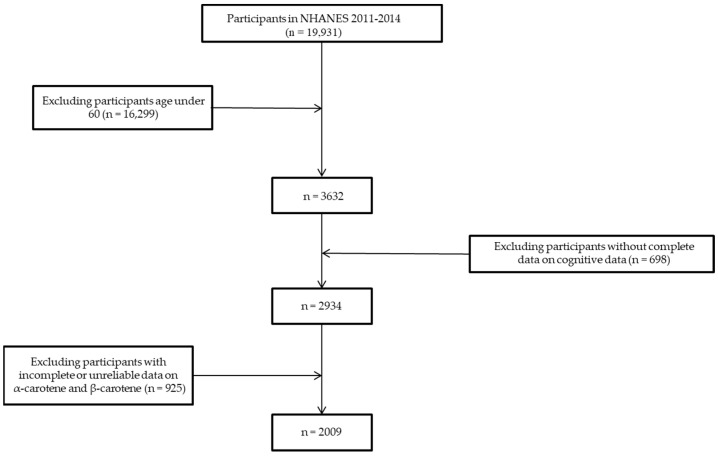
Flow chart for the selection of eligible participants for this study.

The data on dietary α-carotene and β-carotene intake and cognitive function measures were obtained from two cycles of the 2011–2012 and 2013–2014 NHANES. A total of 19,931 participants were recruited in the first round. We then excluded the participants who were under 60 years old (*n* = 16,229) and did not have complete data for cognitive function measurements (*n* = 2934). To reduce the influences of outliers on the analysis, participants with extreme values (dietary α-carotene intake > 1379.5 mcg/d and dietary β-carotene intake > 7876 mcg/d) (*n* = 410) and incomplete 24 h recall data for α-carotene or β-carotene intake (*n* = 515) were also excluded. Finally, 2009 participants were included in this analysis (Figure 1).

### 2.2. Measurement of the Dietary α-Carotene and β-Carotene Intake

The dietary interview component in the NHANES was conducted by the U.S. Department of Agriculture (USDA) and the Department of Health and Human Services (DHHS). Under the cooperation, the survey sample design and data collection were processed by the NCHS. The dietary methodology was designed by the Food Surveys Research Group (FSRG). There were standardized investigation processes and strict quality controls in the survey.

Regarding previous studies, dietary information about α-carotene and β-carotene was obtained from 24 h dietary recall interviews [9,18]. The first day of diet recall was gathered in person during the visit and the next interview was conducted after 3–10 days through telephone. The detailed process of dietary data collection can be found on the NHANES website [19]. Nutrient intake profiles reported by individuals assembled detailed information about various food/beverage items. Information on recalls was acquired from “Total Nutrient Intakes Files”. In this study, the total α-carotene and β-carotene intake was calculated by the mean value of two dietary recalls. We categorized dietary α-carotene and β-carotene intake into quartiles according to published articles for further study (Q1: <25th percentile, Q2: ≥25th to 50th percentile, Q3: ≥50th to 75th percentile, Q4: ≥75th percentile with Q1 as the reference) [20].

### 2.3. Assessments of Cognitive Function

The NHANES survey contained three cognitive function tests including the Consortium to Establish Registry for Alzheimer’s disease (CERAD W-L), Animal Fluency Test (AFT), and Digit Symbol Substitution Test (DSST). These cognitive function tests have been performed in large epidemiological and clinical studies [21,22,23]. For quality control, participants could choose their familiar language during the surveys, and the surveys were administered by two trained interviewers in the Mobile Examination Center. All tests were completed on the same day.

The CERAD W-L was separated into three successive learning trials and a delayed recall. The delayed word recall did not start until the other two cognitive tests were over. The score for each test ranged from 0 to 10 points. In the learning trials, participants were guided to read 10 unrelated words, and then they were asked to recall as many words as possible according to the sequence of the words. The sequence of words would be changed after each trial. The AFT was used to examine categorical verbal fluency, which is a component of executive function. In this test, participants were asked to name as many animals as possible in one minute, and a correct name was assigned one point. Participants in the DSST were required to finish the corresponding logograms in the 133 boxes in 2 min, and the final score was calculated by the total number of correct answers [24,25,26].

Until now, there were no standard cut-off points in the CERAD W-L, AFT, and DSST. According to previous papers, we classified the scores into quartiles and defined the minimum quartile for each test as the reference group [20,27,28]. Regarding the CERAD W-L, AFT, and DSST scores, the cut-off values were 20, 12, and 33, respectively. Participants whose scores were lower than the corresponding cut-off values in tests were assigned to the low cognitive function group, and other participants were assigned to the normal cognitive function groups.

### 2.4. Other Variables of Interest

The set of covariates was based on previous studies [18,29,30]. We selected socioeconomic status variables, including age, gender (male/female), race/ethnicity (Mexican American, other Hispanic, non-Hispanic white, non-Hispanic black, or other race), marital status (married, widowed, divorced, separated, never married, or living with a partner), education (<high school, high school, or >high school), poverty-income ratio, body mass index (BMI), health behavior variables such as smoking and drinking status, and health factors such as diabetes. The poverty ratio was a concept of social economics that was calculated by dividing family (or individual) income by the poverty guidelines specific to the survey year. Smoking status was divided into two groups (yes/no) based on the item “Have you smoked at least 100 cigarettes in your entire life”. Participants were characterized as alcohol drinkers if they consumed at least 12 alcoholic drinks per year. The history of diabetes was defined by the doctor’s diagnosis (yes, no, or borderline). For MCI/dementia participants, confounding factors were provided by family members in the NHANES project.

### 2.5. Statistical Analysis

In this study, both α-carotene and β-carotene were Ln-transformed due to the skewed distribution. Continuous variables and categorical variables are presented as the mean [standard deviation (SD)] and percentage (%), respectively. The differences between the two groups were examined using the Student’s *t*-test and the chi-squared test.

Furthermore, dietary α-carotene and β-carotene intake were modeled as both continuous (Ln-transformed) and categorical variables to explore the associations with cognitive function by logistic regression. In the logistic regression analysis, the crude model had no adjustment, while model I was adjusted for age, gender, race/ethnicity, and BMI. Model II was then adjusted for all potential confounding factors, including age, race/ethnicity, gender, BMI, education, marital status, poverty-income ratio, smoking status, alcohol intake, and diabetes.

We further performed a stratified analysis by gender to examine the associations of dietary α-carotene and β-carotene intake with the three cognitive function tests. Model II was adjusted for all potential covariates mentioned above.

Additionally, the restricted cubic spline (RCS) was utilized to explore the dose-response relationships of dietary α-carotene and β-carotene intake with cognitive function. There were four knots at the 5th, 25th, 75th, and 95th percentiles of the dietary α-carotene and β-carotene intake after adjusting for all covariates. SPSS (version 24.0) and R (vision 4.0.3, R Foundation for Statistical Computing) were used to analyze the data. A two-tailed *p* value less than 0.05 was considered statistically significant.

## 3. Results

Table 1 shows the basic characteristics of the participants from the NHANES 2011–2014 in three cognitive tests (CERAD W-L, AFT, and DSST), which were categorized into low cognition and normal cognition groups. In all cognitive tests, the participants in the low cognitive function group were older, tended to be non-black, be married, and have lower levels of poverty-income ratio, dietary β-carotene intake, and education. In the AFT, the participants in the low cognition group had lower dietary α-carotene intake than those in the normal cognition group. Moreover, in the CERAD W-L, participants in the low cognition group had lower BMI levels than those in the normal cognition group. Participants in the low cognition group from both the AFT and DSST were mostly alcohol drinkers and had no history of diabetes (all *p* values < 0.05). No significant differences in smoking status were observed between the two groups in all cognitive tests.

The associations between dietary α-carotene and β-carotene intake and cognitive functions by the CERAD W-L, AFT, and DSST are depicted in Table 2. In the CERAD W-L, the odds ratio (OR) values in the crude model of cognitive function with β-carotene intake in the second quartile (Q2), third quartile (Q3), and fourth quartile (Q4) groups were 0.72 [95% confidence interval (CI): 0.53, 0.98], 0.66 (95% CI: 0.50, 0.88) and 0.60 (95% CI: 0.37, 0.69), respectively, compared to those in the first quartile (Q1) group. After adjusting for all covariates in model II, the OR value in the Q4 group for dietary β-carotene intake was 0.63 (95% CI: 0.44, 0.90) compared to the Q1 group.

In the AFT, the OR values in the crude model of cognitive function with β-carotene intake in the Q2, Q3, and Q4 groups were 0.65 (95% CI: 0.47, 0.88), 0.51 (95% CI: 0.38, 0.69), and 0.52 (95% CI: 0.38, 0.71), respectively, compared to those in the Q1 group. Furthermore, after adjusting for all confounding factors in model II, the OR value in the Q4 group for dietary β-carotene intake was 0.66 (95% CI: 0.47, 0.94) compared to those in the Q1 group. 

In the DSST, the OR values in the crude model of cognitive function with β-carotene intake in the Q2, Q3, and Q4 groups were 0.64 (95% CI: 0.47, 0.87), 0.45 (95% CI: 0.34, 0.60), and 0.42 (95% CI: 0.30, 0.57), respectively, compared to those in the Q1 group. In model II, the OR value in the Q3 group for β-carotene intake was 0.67 (95% CI: 0.48, 0.83) compared to the Q1 group. No significant difference between the Q4 and Q1 groups were observed after adjusting for all covariates in the DSST. 

There were no significant associations between dietary α-carotene intake and cognitive function by the CERAD W-L and DSST without or with adjustment for covariates. However, in the AFT, the OR of the cognitive function decline with α-carotene intake in the Q4 group was 0.57 (95% CI: 0.41, 0.80) in the crude model and 0.66 (95% CI: 0.46, 0.94) in the fully adjusted model II.

Stratified analyses were performed to investigate the associations of dietary α-carotene and β-carotene intake with the cognitive function measured by the CERAD W-L, AFT, and DSST among the male and female participants (Table 3). Among the male participants, there were significant associations between dietary α-carotene intake (OR: 0.42, 95% CI: 0.25, 0.71) and β-carotene intake (OR: 0.51, 95% CI: 0.30, 0.85) with the cognitive function measured by the AFT in model II. Among the female participants, the OR of cognitive function measured by the CERAD W-L with the Q3 group for α-carotene intake was 0.55 (95% CI: 0.33, 0.91) compared to those in the Q1 group. Furthermore, the OR value of the cognitive function measured by the CERAD W-L with the Q4 group for β-carotene intake was 0.37 (95% CI: 0.21, 0.64), while the OR of the cognitive function measured by the AFT with the Q3 group for β-carotene intake was 0.58 (95% CI: 0.37, 0.91) in model II. No significant associations were observed between dietary α-carotene and β-carotene intake with the cognitive function measured by the DSST.

## 4. Discussion

With the growing aging population, dementia has become a worldwide problem [4]. There are many nutritional factors related to cognitive decline [31]. High-fat and high-sugar diets affect neurogenesis and neuroplasticity by decreasing hippocampal brain-derived neurotrophic factor (BDNF) [32,33], which is a vital mediator of long-term memory formation. Oxidative stress is another necessary factor in cellular injury and the activation of neuroinflammation during the aging process [34]. A previous study indicated that supplementation with folic acid improved cognitive function in older people [35]. In an animal experiment, folates along with vitamins B6 and B12 were associated with DNA methylation in neurons [36]. In a randomized, double-blind, placebo-controlled, multicenter trial, a diet with probiotic supplements could shift gut microbiota status, which was inversely associated with BDNF levels in the blood among older people [37]. To date, some studies have examined the associations of α-carotene and β-carotene with cognitive function. However, to our knowledge, most of the previous studies have mainly focused on the plasma level of carotenes with cognitive function [15,38,39]. Since the plasma concentrations are generally maintained within a certain range, it may be difficult to explore the effect of actual intake. Creatively, our study explored the associations of dietary α-carotene and β-carotene intake with cognitive performance using the CERAD W-L, AFT, and DSST.

In this research, we found that dietary α-carotene and β-carotene intake were inversely associated with cognitive function decline. β-carotene intake showed negative relationships with cognitive performance in all three cognitive function tests, while α-carotene intake was only negatively associated with cognitive performance in the ATF. In the RCS results, after adjusting for all potential covariates, there were approximately linear dose-response relationships of β-carotene intake with CERAD W-L, AFT, and DSST decline. In previous studies, we found that a high plasma level of α-carotene in patients was associated with higher cognition function scores [15]. A randomized trial study of 4052 participants reported that the participants had a higher global score in the β-carotene intake group than in the control group. In the verbal memory test, men with durable beta carotene replenishment also had significantly better scores than the control group [40]. Another survey of 298 participants also found that serum β-carotene was significantly associated with cognitive function [44]. Perrig et al. found that a higher β-carotene plasma level was associated with better memory performance in 442 participants aged 65–94 years [41]. However, this relationship in the DSST changed when the Ln-transformed dietary β-carotene intake exceeded 8.7 mcg/day, indicating that dietary β-carotene intake may not have positive effects on cognitive function decline. In a meta-analysis, an overdose of β-carotene intake enhanced the mortality of cancers and other diseases [42].

The mechanism of the effect of dietary carotene intake on cognitive decline remains unclear. In a previous paper, the progression of cognitive decline was related to vascular diseases [43]. Dietary carotene intake reduced the progression of atherosclerosis, stroke, and other oxidative impairments, which are risk factors for cognitive decline [43]. Carotenes are mainly obtained through daily food intake [15], so another hypothesis is related to the antioxidant function of plasma α-carotene and β-carotene through dietary intake, which can promote the formation of gap junctions between cells and can be converted to vitamin A [16]. In previous studies, vitamin A had positive effects on human health, including cognitive function and neurodevelopment [39,45,46]. In contrast, long-term vitamin A deficiency could cause cognitive function decline by affecting the nuclear receptors RXR and RAR (mainly present in the hippocampus, cortex, and caudate) to initiate target gene transcription [47]. Furthermore, a survey of 2983 participants indicated that carotenoid-rich dietary patterns containing α-carotene and β-carotene were associated with better cognitive function consequences in older people [38]. In addition, lutein and zeaxanthin, as parts of carotenoids, can also improve cognitive function [48,49] in either older or younger generations [50,51]. Moreover, dietary foods containing α-carotene and β-carotene are full of vitamin E, selenium, and flavonoids [52] which have benefits on cognitive function decline [53,54,55]. Thus, we cannot exclude the effects of other nutrients in food on cognitive decline without laboratory experiments.

We found differences in cognitive function between genders in our study, which may be related to hormones. A previous study found that levels of plasma β-carotene in men were lower than in women in each tertile of daily intake of fruits and vegetables [56]. In a previous animal experiment, the efficiency of β-carotene conversion to vitamin A in female rats was higher than that in male rats and the difference was related to hormone-regulated genes [57].

This study has several advantages. First, we used a large-scale sample of older adults in the United States. Second, the quality of data in the NHANES could be guaranteed in terms of survey methods and quality control. In addition, we controlled a wide range of potential confounders to estimate the associations of dietary α-carotene and β-carotene intake with cognitive function decline. Our study also contains several limitations. First, due to the cross-sectional nature of the study, we cannot confirm the causal relationships between dietary α-carotene and β-carotene intake and cognitive function. Moreover, although we controlled basic social confounding of carotenes and cognitive function, not all possible confounding factors were included. Furthermore, since the functions of dietary α-carotene on the CERAD W-L and DSST were not observed, further studies are still needed. Finally, the levels of dietary α-carotene and β-carotene intake were collected through the questionnaire survey rather than the measurement in the laboratory. An RCT study combined with laboratory confirmations is still needed to explore more accurate relationships of dietary α-carotene and β-carotene intake with cognitive function in the future.

## 5. Conclusions

In this study, our results reflected that dietary α-carotene and β-carotene intake might have inverse effects on cognitive function decline in older people. However, the excessive intake of dietary α-carotene and β-carotene may be a problem that needs special attention. Further longitudinal studies and laboratory confirmations are required to confirm these results.

## Figures and Tables

**Figure 2:**
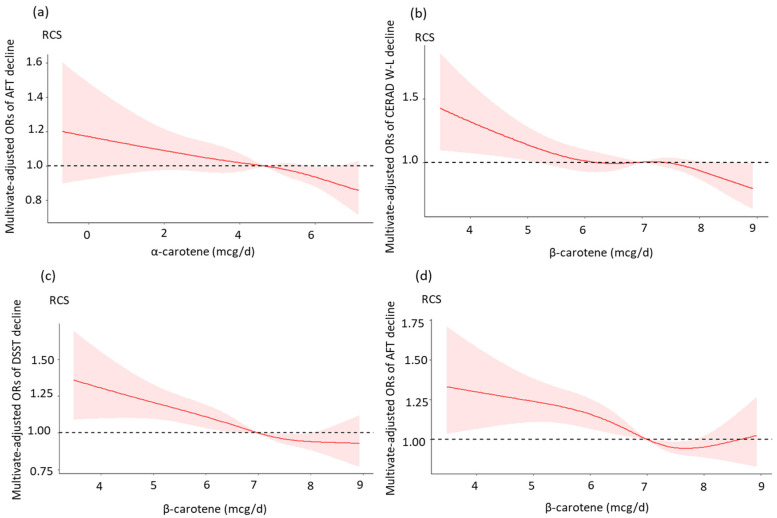
Dose–response relationships between dietary α-carotene and β-carotene intake and cognitive performance. (**a**) Restricted cubic spline model of the ORs of the AFT decline with Ln-transformed dietary α-carotene intake; (**b**) Restricted cubic spline model of the ORs of the CERAD W-L decline with Ln-transformed dietary β-carotene intake; (**c**) Restricted cubic spline model of the ORs of the DSST decline with Ln-transformed dietary β-carotene intake; (**d**) Restricted cubic spline model of the ORs of the AFT decline with Ln-transformed dietary β-carotene intake. All models were adjusted for age, gender, race/ethnicity, BMI, education, marital status, poverty-income ratio, smoking status, alcohol intake, and diabetes.

**Table 1:** Baseline characteristics of participants classified by cognitive function status.

	CERAD W-L			AFT			DSST		
	Low Cognitive Function	Normal Cognitive Function	*p*-Value	Low Cognitive Function	Normal Cognitive Function	*p*-Value	Low Cognitive Function	Normal Cognitive Function	*p*-Value
**Age ^a^**	72.22 (6.84)	68.56 (6.44)	**<0.001**	71.17 (6.82)	68.95 (6.61)	**<0.001**	71.61 (6.81)	68.79 (6.56)	**<0.001**
**Poverty-income ratio ^a^**	2.23 (1.51)	2.76 (1.59)	**<0.001**	2.16 (1.46)	2.77 (1.59)	**<0.001**	1.78 (1.26)	2.89 (1.58)	**<0.001**
**BMI ^a^**	28.73 (6.40)	29.70 (6.53)	**0.005**	29.25 (6.39)	29.53 (6.54)	0.424	29.46 (6.52)	29.47 (6.51)	0.974
**Ln α-carotene ^a^**	4.49 (1.67)	4.60 (1.62)	0.218	4.40 (1.59)	4.62 (1.64)	**0.013**	4.48 (1.63)	4.60 (1.63)	0.155
**Ln β-carotene ^a^**	6.62 (1.23)	6.92 (1.08)	**<0.001**	6.61 (1.22)	6.91 (1.09)	**<0.001**	6.54 (1.23)	6.94 (1.07)	**<0.001**
**Gender ^b^**									
male	291 (60.4%)	694 (45.4%)	**<0.001**	219 (49.8%)	766 (48.8%)	0.724	254 (55.0%)	731 (47.3%)	**0.004**
female	191 (39.6%)	833 (54.6%)		221 (50.2%)	803 (51.2%)		208 (45.0%)	816 (52.7%)	
**Race/ethnicity ^b^**									
Mexican American	52 (10.8%)	113 (7.4%)	**0.035**	34 (7.7%)	131 (8.3%)	**<0.001**	61 (13.2%)	104 (6.7%)	**<0.001**
Other Hispanic	55 (11.4%)	134 (8.8%)		48 (10.9%)	141 (9.0%)		81 (17.5%)	108 (7.0%)	
Non-Hispanic White	239 (49.6%)	804 (52.7%)		162 (36.8%)	881 (56.2%)		152 (32.9%)	891 (57.6%)	
Non-Hispanic Black	107 (22.2%)	356 (23.3%)		157 (35.7%)	306 (19.5%)		155 (33.5%)	308 (19.9%)	
Other Race	29 (6.0%)	120 (7.9%)		39 (8.9%)	110 (7.0%)		13 (2.8%)	136 (8.8%)	
**Marital status ^b^**									
Married	256 (53.1%)	869 (56.9%)	**0.004**	224 (50.9%)	901 (57.4%)	**<0.001**	214 (46.3%)	911 (58.9%)	**<0.001**
Widowed	114 (23.7%)	262 (17.2%)		115 (26.1%)	261 (16.6%)		127 (27.5%)	249 (16.1%)	
Divorced	53 (11.0%)	234 (15.3%)		58 (13.2%)	229 (14.6%)		55 (11.9%)	232 (15.0%)	
Separated	16 (3.3%)	44 (2.9%)		17 (3.9%)	43 (2.7%)		29 (6.3%)	31 (2.0%)	
Never married	25 (5.2%)	84 (5.5%)		19 (4.3%)	90 (5.7%)		25 (5.4%)	84 (5.4%)	
Living with a partner	18 (3.7%)	34 (2.2%)		7 (1.6%)	45 (2.9%)		12 (2.6%)	40 (2.6%)	
**Education ^b^**									
<High school	187 (38.8%)	275 (18.0%)	**<0.001**	158 (35.9%)	304 (19.4%)	**<0.001**	240 (51.9%)	222 (14.4%)	**<0.001**
High school	115(23.9%)	368 (24.1%)		128 (29.1%)	355 (22.6%)		114 (24.7%)	369 (23.9%)	
>High school	180 (37.3%)	884 (57.9%)		154 (35.0%)	910 (58.0%)		108 (23.4%)	956 (61.8%)	
**Smoking status ^b^**									
Yes	243 (50.4%)	803 (52.6%)	0.405	232 (52.7%)	814 (51.9%)	0.753	238 (51.5%)	808 (52.2%)	0.787
No	239 (49.6%)	724 (47.4%)		208 (47.3%)	755 (41.8%)		224 (48.5%)	739 (47.8%)	
**Alcohol intake ^b^**									
Yes	334 (69.3%)	1074 (70.3%)	0.664	287 (65.2%)	1121 (71.4%)	**0.012**	293 (63.4%)	1115 (72.1%)	**<0.001**
No	148 (30.7%)	453 (29.7%)		153 (34.8%)	448 (28.6%)		169 (36.6%)	432 (27.9%)	
**Diabetes ^b,c^**									
Yes	132 (27.4%)	353 (23.1%)	0.161	136 (30.9%)	349 (22.2%)	**0.001**	153 (33.1%)	332 (21.5%)	**<0.001**
No	328 (68.0%)	1101 (72.1%)		286 (65.0%)	1143 (72.8%)		288 (62.3%)	1141 (73.8%)	
Borderline	22 (6.4%)	73 (4.8%)		18 (4.1%)	77 (4.9%)		21 (4.5%)	74 (4.8%)	

Note: ^a^ mean (SD), ^b^ *n* (%). ^c^ Diabetes variable was defined by whether a person had been told by a doctor or other health professional that they had diabetes or borderline diabetes.

**Table 2:** Weighted odds ratio (95% confidence intervals) of low cognitive performance by quartiles of dietary α-carotene and β-carotene intake.

	CERAD W-L			AFT			DSST		
	Crude	Model I ^a^	Model II ^b^	Crude	Model I ^a^	Model II ^b^	Crude	Model I ^a^	Model II ^b^
**α-carotene** (mcg/d)									
Q1 (≤17.5)	Reference	Reference	Reference	Reference	Reference	Reference	Reference	Reference	Reference
Q2 (17.5 to ≤62.5)	0.85 (0.62, 1.16)	0.79 (0.56, 1.09)	0.89 (0.63, 1.25)	0.85 (0.62, 1.17)	0.83 (0.60, 1.14)	0.95 (0.68, 1.33)	1.00 (0.72, 1.37)	0.93 (0.67, 1.29)	1.26 (0.87, 1.83)
Q3 (62.5 to ≤358)	0.74 (0.55, 1.00)	0.69 (0.50, 0.94) *	0.82 (0.59, 1.14)	0.86 (0.63, 1.16)	0.81 (0.59, 1.10)	0.98 (0.71, 1.35)	0.84 (0.62, 1.13)	0.77 (0.56, 1.06)	1.16 (0.81, 1.65)
Q4 (>358)	0.79 (0.58, 1.09)	0.70 (0.50, 0.97) *	0.84 (0.60, 1.19)	0.57 (0.41, 0.80) *	0.53 (0.38, 0.74) **	0.66 (0.46, 0.94) *	0.80 (0.58, 1.11)	0.73 (0.52, 1.02)	1.15 (0.79, 1.69)
**β-carotene** (mcg/d)									
Q1 (≤338)	Reference	Reference	Reference	Reference	Reference	Reference	Reference	Reference	Reference
Q2 (338 to ≤819)	0.72 (0.53, 0.98) *	0.67 (0.49, 0.93) *	0.84 (0.60, 1.18)	0.65 (0.47, 0.88) *	0.65 (0.47, 0.89) *	0.80 (0.57, 1.10)	0.64 (0.47, 0.87) *	0.60 (0.44, 0.81) *	0.89 (0.63, 1.26)
Q3 (819 to ≤2222.5)	0.66 (0.50, 0.88) **	0.61 (0.46, 0.83) *	0.81 (0.59, 1.11)	0.51 (0.38, 0.69) **	0.49 (0.36, 0.66) **	0.65 (0.48, 0.89) *	0.45 (0.34, 0.60) **	0.42 (0.31, 0.56) **	0.67 (0.48, 0.83) *
Q4 (>2222.5)	0.60 (0.37, 0.69) **	0.46 (0.33, 0.64) **	0.63 (0.44, 0.90) *	0.52 (0.38, 0.71) **	0.48 (0.35, 0.66) **	0.66 (0.47, 0.94) *	0.42 (0.30, 0.57) **	0.39 (0.28, 0.54) **	0.73 (0.50, 1.06)

^a^ Model I was adjusted for age, gender, race/ethnicity, and BMI. ^b^ Model II was adjusted for age, gender, race/ethnicity, BMI, education, marital status, poverty-income ratio, smoking status, alcohol intake, and diabetes. * *p* < 0.05, ** *p* < 0.01.

**Table 3:** Weighted odds ratio (95% confidence intervals) of low cognitive performance by quartiles of dietary α-carotene and β-carotene intake, stratified by gender.

	CERAD W-L		AFT		DSST	
	Crude	Model II ^a^	Crude	Model II ^a^	Crude	Model II ^a^
**Male**						
**α-carotene** (mcg/d)						
Q1 (≤17.5)	Reference	Reference	Reference	Reference	Reference	Reference
Q2 (17.5 to ≤62.5)	0.97 (0.64, 1.47)	1.02 (0.66, 1.60)	0.87 (0.57, 1.33)	0.96 (0.61, 1.51)	1.04 (0.69, 1.57)	1.31 (0.81, 2.12)
Q3 (62.5 to ≤358)	0.95 (0.64, 1.42)	1.10 (0.71, 1.69)	0.84 (0.55, 1.27)	1.00 (0.64, 1.56)	0.69 (0.46, 1.04)	0.98 (0.61, 1.59)
Q4 (>358)	0.89 (0.59, 1.34)	0.98 (0.62, 1.53)	0.37 (0.23, 0.60) **	0.42 (0.25, 0.71) **	0.62 (0.40, 0.96) *	0.89 (0.54, 1.47)
**β-carotene** (mcg/d)						
Q1 (≤338)	Reference	Reference	Reference	Reference	Reference	Reference
Q2 (338 to ≤819)	0.84 (0.55, 1.26)	1.05 (0.67, 1.63)	0.72 (0.47, 1.10)	0.95 (0.60, 1.50)	0.61 (0.40, 0.92) *	0.94 (0.58, 1.51)
Q3 (819 to ≤2222.5)	0.79 (0.54, 1.17)	0.99 (0.65, 1.51)	0.57 (0.38, 0.85) **	0.73 (0.47, 1.13)	0.46 (0.31, 0.68) **	0.68 (0.43, 1.07)
Q4 (>2222.5)	0.71 (0.46, 1.08)	0.94 (0.59, 1.51)	0.41 (0.25, 0.65) **	0.51 (0.30, 0.85) **	0.35 (0.22, 0.55) **	0.64 (0.38, 1.09)
**Female**						
**α-carotene** (mcg/d)						
Q1 (≤17.5)	Reference	Reference	Reference	Reference	Reference	Reference
Q2 (17.5 to ≤62.5)	0.74 (0.45, 1.21)	0.70 (0.41, 1.20)	0.85 (0.52, 1.39)	0.94 (0.56, 1.57)	1.01 (0.60, 1.71)	1.22 (0.68, 2.23)
Q3 (62.5 to ≤358)	0.61 (0.38, 0.97) *	0.55 (0.33, 0.91) *	0.91 (0.58, 1.43)	0.98 (0.60, 1.58)	1.15 (0.71, 1.86)	1.39 (0.83, 2.41)
Q4 (>358)	0.72 (0.44, 1.17)	0.66 (0.39, 1.13)	0.86 (0.53, 1.40)	0.99 (0.59, 1.65)	1.17 (0.71, 1.95)	1.57 (0.87, 2.83)
**β-carotene** (mcg/d)						
Q1 (≤338)	Reference	Reference	Reference	Reference	Reference	Reference
Q2 (338 to ≤819)	0.59 (0.37, 0.94) *	0.64 (0.39, 1.06)	0.58 (0.37, 0.91) *	0.65 (0.40, 1.04)	0.67 (0.43, 1.06)	0.83 (0.50, 1.40)
Q3 (819 to ≤2222.5)	0.55 (0.36, 0.83) **	0.63 (0.40, 1.00)	0.46 (0.31, 0.70) **	0.58 (0.37, 0.91) *	0.45 (0.30, 0.69) **	0.66 (0.41, 1.08)
Q4 (>2222.5)	0.33 (0.20, 0.54) **	0.37 (0.21, 0.64) **	0.63 (0.41, 0.97) *	0.81 (0.50, 1.30)	0.50 (0.32, 0.79) **	0.83 (0.49, 1.41)

^a^ Model II was adjusted for age, race/ethnicity, BMI, education, marital status, poverty-income ratio, smoking status, alcohol intake, and diabetes. * *p* < 0.05, ** *p* < 0.01. Figure 2 presents the results of the restricted cubic spline analysis. For the dietary α-carotene intake, there was a linear dose-response relationship between Ln-transformed α-carotene and the AFT decline. Ln-transformed dietary β-carotene intake was significantly associated with a decreased probability of CERAD W-L decline, showing a reduced possibility of AFT decline and DSST decline (all nonlinear *p* > 0.05). However, when the Ln-transformed dietary β-carotene intake reached 8.7 mcg/day, the relationship was no longer significant. Furthermore, there were no significant associations between dietary α-carotene intake and cognitive function assessed by the CERAD W-L and DSST, and the results are not shown in the following graph.

## Data Availability

The data are publicly available on the NHANES website: https://www.cdc.gov/nchs/nhanes/Index.htm (accessed on 10 June 2022).
